# Dual-task related frontal cerebral blood flow changes in older adults with mild cognitive impairment: A functional diffuse correlation spectroscopy study

**DOI:** 10.3389/fnagi.2022.958656

**Published:** 2022-12-20

**Authors:** Cristina Udina, Stella Avtzi, Miriam Mota-Foix, Andrea L. Rosso, Joan Ars, Lisa Kobayashi Frisk, Clara Gregori-Pla, Turgut Durduran, Marco Inzitari

**Affiliations:** ^1^REFiT Barcelona Research Group, Parc Sanitari Pere Virgili and Vall d’Hebron Research Institute (VHIR), Barcelona, Spain; ^2^Medicine Department, Universitat Autònoma de Barcelona, Barcelona, Spain; ^3^ICFO – Institut de Ciències Fotòniques, The Barcelona Institute of Science and Technology, Barcelona, Spain; ^4^Statistics and Bioinformatics Unit, Vall d’Hebron Institut de Recerca (VHIR), Barcelona, Spain; ^5^Department of Epidemiology, School of Public Health, University of Pittsburgh, Pittsburgh, PA, United States; ^6^Institució Catalana de Recerca i Estudis Avançats (ICREA), Barcelona, Spain; ^7^Faculty of Health Sciences, Universitat Oberta de Catalunya (UOC), Barcelona, Spain

**Keywords:** mild cognitive impairment, dual-task (DT), prefrontal cortex, cerebral blood flow, spectroscopy, aging

## Abstract

**Introduction:**

In a worldwide aging population with a high prevalence of motor and cognitive impairment, it is paramount to improve knowledge about underlying mechanisms of motor and cognitive function and their interplay in the aging processes.

**Methods:**

We measured prefrontal cerebral blood flow (CBF) using functional diffuse correlation spectroscopy during motor and dual-task. We aimed to compare CBF changes among 49 older adults with and without mild cognitive impairment (MCI) during a dual-task paradigm (normal walk, 2- forward count walk, 3-backward count walk, obstacle negotiation, and heel tapping). Participants with MCI walked slower during the normal walk and obstacle negotiation compared to participants with normal cognition (NC), while gait speed during counting conditions was not different between the groups, therefore the dual-task cost was higher for participants with NC. We built a linear mixed effects model with CBF measures from the right and left prefrontal cortex.

**Results:**

MCI (*n* = 34) showed a higher increase in CBF from the normal walk to the 2-forward count walk (estimate = 0.34, 95% CI [0.02, 0.66], *p* = 0.03) compared to participants with NC, related to a right- sided activation. Both groups showed a higher CBF during the 3-backward count walk compared to the normal walk, while only among MCI, CFB was higher during the 2-forward count walk.

**Discussion:**

Our findings suggest a differential prefrontal hemodynamic pattern in older adults with MCI compared to their NC counterparts during the dual-task performance, possibly as a response to increasing attentional demand.

## 1 Introduction

The aging of the worldwide population and the consequent increase in dementia and disability makes it crucial to improve knowledge about the pathophysiology underlying cognitive and motor impairments to establish preventive and treatment strategies to minimize related negative outcomes. Cognitive and motor impairments, both independently and concomitantly, are prevalent among older adults and have a negative impact on health-related outcomes, including increased healthcare costs and worsening quality of life (Verghese et al., 2006; Callisaya et al., 2011; Gaugler et al., 2016; Barbe et al., 2018). Pre-dementia states, such as mild cognitive impairment (MCI) or motoric cognitive risk syndrome, have been linked to a higher risk of falls and disability besides dementia (Verghese et al., 2008; Farias et al., 2009; Callisaya et al., 2016; Doi et al., 2017). In this context, there is growing evidence that supports the notion of a motor-cognitive interplay. For example, slowing gait speed predicted cognitive impairment in a longitudinal study of cognitively healthy subjects at the study’s baseline (Rosso et al., 2017), and cognitive function, in particular executive function, has been associated with gait speed (Martin et al., 2013; Callisaya et al., 2014). This interplay is further supported by a common neural substrate since the prefrontal cortex (PFC) is involved in the neural control of gait and cognitive function. In particular, executive function (Alvarez and Emory, 2006; Lezak, 2012; Mirelman et al., 2018) is involved in gait control (Holtzer et al., 2006; Yogev-Seligmann et al., 2008). Furthermore, when a cognitive task is performed during a motor task, the dual-task performance declines relative to performance in each individual task, which is known as dual-task interference (Allali et al., 2007; Hausdorff et al., 2008; Montero-Odasso et al., 2012). The decrement in dual-task performance compared to the single task is larger with increasing cognitive demand (Allali et al., 2007), as well as in people with impaired mobility (Bloem et al., 2006) or cognition, especially executive dysfunction (Hausdorff et al., 2008). It seems that both tasks may compete for the same neural resources including, but not exclusively, the PFC (Collette et al., 2005; Leone et al., 2017), but the underlying neural mechanisms remain unclear. Prior studies have demonstrated that dual-task paradigms with a higher cognitive load may help differentiate individuals with MCI from their healthy counterparts (Bahureksa et al., 2017; Bishnoi and Hernandez, 2021), which may be due to competition for neural resources and reduced availability of resources in those with MCI.

Neuroimaging is an essential tool to further understand these factors, and functional neuroimaging studies have traditionally used functional magnetic resonance imaging (fMRI) and positron emission tomography, among other methods, to assess changes in brain activity related to different cognitive stimuli (Gourovitch et al., 2000; Jurado and Rosselli, 2007; Cui et al., 2011). As an attempt to study gait neural pathways, some studies have applied these techniques during the imagined walk (Blumen et al., 2014). Recently, the introduction of diffuse optical techniques, such as functional near-infrared spectroscopy (fNIRS), has allowed the study of brain activation during the execution of motor tasks, including dual-task walking (Holtzer et al., 2014). Other advantages of diffuse optics include its non-invasiveness, relatively low cost, portability, and, contrary to fMRI, suitability for patients with pacemakers, metallic implants, or those suffering from claustrophobia. Notably, an increasing body of literature from fNIRS studies supports its role in this area (Agbangla et al., 2017; Gramigna et al., 2017). Evidence from fNIRS studies suggests a different pattern in PFC oxygenation among older adults with MCI during walking under challenging circumstances compared to cognitively healthy counterparts (Udina et al., 2020).

A relatively more recent method, diffuse correlation spectroscopy (DCS) also uses near-infrared light to measure microvascular cerebral blood flow (CBF). Both fNIRS and DCS techniques use near-infrared light to obtain information about cerebral hemodynamics in a very similar manner. By using optical fibers placed on the forehead, light is emitted on the tissue and later the diffused light is collected by detector fibers as well. However, the principles and methodological analysis are different. fNIRS modality provides information about tissue oxygen metabolism through measures of oxygenated and deoxygenated hemoglobin concentrations. On the other hand, DCS techniques can provide information about the local microvascular blood flow of the measured area by assessing red blood cell movement based on laser speckle statistics (Boas et al., 1995; Boas and Yodh, 1997; Durduran et al., 2010; Mesquita et al., 2011). According to the neurovascular coupling phenomenon, an increase in oxygen consumption to meet energy demands in activated cerebral areas would cause an increase in local blood flow, resulting in an increase in oxyhemoglobin and a decrease in deoxyhemoglobin. Underlying mechanisms consist of complex regulatory pathways that need further research (Phillips et al., 2016). The use of functional DCS (fDCS) might provide relevant information about this phenomenon since measures of CBF might complement information regarding oxygen metabolism provided by fNIRS, and hence provide insight into brain metabolism and neurovascular coupling. In addition, a great advantage of fDCS lies in the fact that the technique is more sensitive to the intracranial signal compared to extracerebral noise, as reported by Selb et al. (2014). Previous studies have validated DCS modality against standard techniques in humans and animals such as arterial-spin labeled (Yu et al., 2007; Milej et al., 2020), magnetic resonance imaging, transcranial doppler ultrasound (Buckley et al., 2009; Busch et al., 2016), positron emission tomography (Giovannella et al., 2020), and others. In addition, DCS has been used to study both inpatient and outpatient populations ranging from pediatric patients to adult neurocritical care patients (Durduran et al., 2004; Jaillon et al., 2007; Jain et al., 2014; Roche-Labarbe et al., 2014; Li et al., 2018). fDCS shares the same features as fNIRS and, therefore, could be used during motion. However, to our knowledge, the study of brain activity during gait and the dual-task with fDCS has never been reported. Assessing CBF changes during gait may complement evidence of changes in oxygenation from fNIRS studies, hence contributing to the understanding of neural mechanisms of gait provided by fNIRS studies.

The global aim of the study was to assess CBF changes in the PFC with fDCS among high-functioning, community-dwelling older adults with and without MCI during dual-task walking under various degrees of attention-demanding load. In particular, we sought to investigate whether (1) CBF changes were higher during various dual-task conditions compared to normal walk (NW); (2) CBF changes from NW to a dual-task differ between the cognitive status group [MCI vs. normal cognition (NC)], and (3) various clinical covariates affect CBF patterns. For the dual-task paradigm, we chose three dual-task (forward counting, backward counting, and obstacle negotiation) to assess the impact of different kinds of secondary tasks (Al-Yahya et al., 2011; Bishnoi and Hernandez, 2021) and increased attentional demand with two counting tasks, i.e., backward being more challenging than forward counting (Schwenk et al., 2010). We also included a heel tapping task as a rhythmic motor task that did not involve gait as we speculated it could be linked to different PFC hemodynamic changes than gait. Due to neurovascular coupling, one could speculate that the higher activation related to oxygenation changes shown in fNIRS studies during the dual-task (Doi et al., 2013; Holtzer et al., 2015; Chaparro et al., 2017) should be linked to a higher CBF (Phillips et al., 2016). Hence, we hypothesized that CBF would be higher during the dual-task compared to NW and that the CBF change from NW to the dual-task would be higher among MCI compared to persons with NC.

## 2 Materials and methods

### 2.1 Study design

A cross-sectional observational study was conducted among community-dwelling older adults with MCI and their counterparts with NC (the MEDPHOTAGE study).

### 2.2 Setting and participants

A convenience sample of patients from the outpatient memory clinic of Parc Sanitary Pere Virgili (Barcelona, Spain) was enrolled if they met the following criteria: ≥65 years old, having preserved function for activities of daily living, and being able to walk at least 50 m without assistance (walking aid devices, including cane or crutch, were accepted), according to self-reported information provided by the candidate and corroborated by relatives, when appropriate. The exclusion criteria included illiteracy, uncorrected significant visual or auditory impairment, dementia, overt psychiatric or neurological disease despite appropriate drug therapy (depression, delirium, stroke, and Parkinson’s disease), cardiopulmonary disease that is not well controlled with medication, functional classification III-IV of the New York Heart Association (Dolgin, 1994) and/or the need for oxygen therapy, being terminally ill with a life expectancy less than 6 months, and current use of neuroleptics or anticonvulsants.

Demographical and clinical variables as well as neuropsychological assessment were collected at inclusion in the outpatient clinic. Then, the participants were scheduled for two separate assessments. Due to the long CBF measurement protocol that lasted around 2 h, we decided to separate the CBF measurements from the physical performance assessment (Section “2.5 Physical performance assessment”).

### 2.3 Cognitive impairment diagnosis

A MCI diagnosis was determined in the outpatient clinic’s case conference after an expert assessment by a geriatrician and a neuropsychologist following well-established criteria (Petersen, 2004). Briefly, MCI was identified if cognitive complaints were corroborated by scores in neuropsychological tests below the normal range (adjusted for age and years of education) and if the person maintained preserved activities of daily living or with functional impairment not due to cognitive complaints. Cognitive domains assessed included global cognition (MMSE), memory (Rey auditory verbal learning test), working memory (digit span), phonemic and semantic verbal fluency, symbol digit modalities test, language (Boston Naming Test), constructive apraxia (Wechsler Adult Intelligence Scale 4th edition), ideomotor and ideational apraxia (Luria test), visual perception (Poppelreuter overlapping figures), and visuospatial recognition (Luria test). A classification into four clinical subtypes has been proposed depending on whether memory is impaired and whether there is impairment in one (single-domain) or several cognitive domains (multi-domain), such as executive functions, language, visuospatial skills, etc. In our study, MCI was classified into two groups: single-domain and multi-domain MCI following those criteria.

Patients from the outpatient clinic without cognitive impairment after clinical assessment and relatives of participants without cognitive complaints were assessed for inclusion. Participants had to score 27 or higher in the mini-mental state exam (MMSE) to be included in the NC group.

Dementia was ruled out of the outpatient memory clinic’s case conference. Information regarding the performance of the activities of daily living was extracted during the interview from the participant and caregiver.

The research protocol’s procedures were according to the Declaration of Helsinki and were approved by the local ethics committee (Universitat Autònoma de Barcelona, Spain). All participants provided written informed consent.

### 2.4 Assessment of cerebral blood flow

#### 2.4.1 Dual-task paradigm

The measurements were performed in a quiet, well-lit room with an 8-m walkway. The researchers explained the whole protocol before the start of the first resting period, and a short instruction for each task was given after the rest period and immediately before the start of the corresponding task. The participants were asked to walk back and forth five loops, i.e., 16 m each, over the walkway for each of the four walking tasks that are described below. The total walking distance was 80 m (5 m × 16 m). A single trial of each task was performed. Importantly, the participants were instructed to walk at a self-selected pace and not to prioritize either of the tasks while performing the dual-task (Verghese et al., 2007). The researchers did not interact with the participants except to provide instructions or to assist the participant if necessary.

**FIGURE 1:**
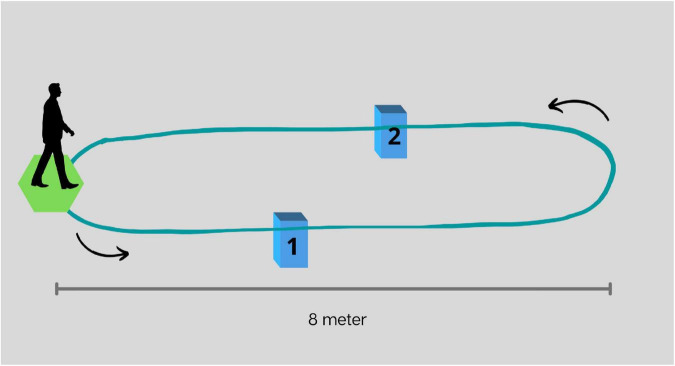
Representation of the walkway and placement of obstacles for walk while negotiating obstacles. The first obstacle (1), 15 cm in height, and placed at a 4-m distance from the start of the walking course, and the second (2), 10 cm in height, and placed at a 6-m distance.

Instructions were provided as follows:

• Normal walk: Participants were instructed to walk five loops over the 8-m walkway.
• Walk while 2-forward counting (FWC): Participants were asked to perform serial 2-forward calculations (e.g., 2, 4, 6,…) while walking five loops over the walkway.
• Walk while 3-backward counting (BWC): Participants were asked to perform serial 3-backward calculations (e.g., 53, 50, 47,…) while walking five loops over the walkway.
• Walk while negotiating obstacles (WWO): Participants were instructed to walk over two small obstacles placed on the walkway. The first obstacle (15 cm of height) was placed at a 4-m distance from the start of the walking course and the second (10 cm of height) at a 6-m distance. See Figure 1.

• Heel tapping (TAP) task: Participants were instructed to alternatively elevate each heel while seated for 1 min.

**FIGURE 2:**
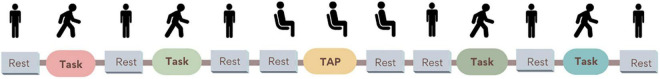
Dual-task paradigm during cerebral blood flow (CBF) measures. Rest periods: participants were instructed to remain silent and refrain from moving while standing. Only before and after the heel tapping task *(TAP)*, participants rested in a sitting position to avoid CBF changes related to orthostatic changes. Walking tasks (normal walk, walk while 2-forward count, walk while 3-backward count, and walk while negotiating obstacles) order was randomized to minimize the fatigue effect.

Baseline CBF was assessed during resting periods before and after each task, where participants were instructed to avoid moving and talking. To avoid orthostatic changes right before the tasks, rest periods before and after walking tasks were performed standing, and resting periods before and after TAP were performed sitting on a chair. The first rest period lasted 2 min while the remaining rest periods lasted 1.5 min. The order of walking tasks was randomized; however, TAP was consistently the third test to minimize the possible effect of sitting rest in the following tasks. See Figure 2.

For the behavioral results, we recorded the time required to walk five loops over the 8-m walkway using a stopwatch. We report gait speed (m/sec) during each walking task [80 m/duration (s)] and dual-task cost for each task as previously described [((dual-task−single task)/single task) × 100] (Beurskens and Bock, 2012). It is worth noting that the gait assessment included turns to perform loops over the walkway, so the gait speed reported here is not limited to a steady-state walk. For the cognitive output, research assistants identified counting errors as a categorical variable. When the participant miscalculated more than three calculations, “counting errors” were identified as “Yes.” Participants who were not able to perform the backward calculations while walking were instructed to stop the BWC trial.

#### 2.4.2 Functional diffuse correlation spectroscopy system and physiological data acquisition

The optical data collection was performed with a custom-made DCS system (Durduran et al., 2010; Durduran and Yodh, 2014) with a temporal resolution of 9 s. Due to an early technical issue with the device, 6 s of the acquisition was discarded. In other words, 3 s of DCS data was acquired with a 6-s off-period in-between. This issue was identified after several subjects were measured, and to be able to keep all data comparable, we opted not to resolve it.

The device utilized probes suitable for placement over the prefrontal lobes for independent measurements of the CBF from the two hemispheres. DCS derives a blood flow index (BFI) corresponding to microvascular CBF in the probe region that extends to the top of the cerebral cortex. The details of the technique and the instrumentation were previously published (Durduran et al., 2010; Durduran and Yodh, 2014; Gregori Pla, 2019). The device was further customized for this study. An uninterruptible power supply (K-LCD 1200, Protec-Sai, Barcelona, Spain) allowed the device to be unplugged for the motor tasks to follow each participant without disturbing their performance during the instructed tasks.

In addition to DCS, a capnography (Capnostream™ 20p, Medtronic, United States) was used to record the respiratory and systemic parameters since they are known to influence diffuse optical signals. In particular, we synchronized the capnography to the DCS and obtained end-tidal carbon dioxide (CO_2_) concentration, peripheral arterial oxygen saturation, and heart rate continuously. Finally, an accelerometer was also placed on the head of each participant to record the motion and to regress out potential motion artifacts during the tasks.

**FIGURE 3:**
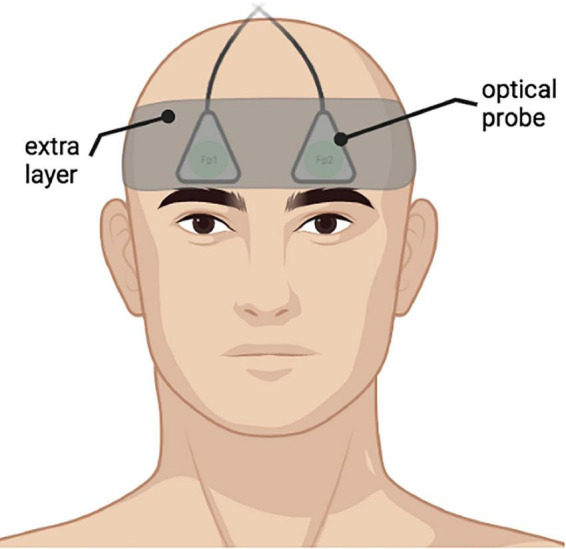
An example of placement of the optical probes on a participant bilaterally on Fp1, Fp2 positions according to “10–20 system”. An extra fabric layer was applied on top of the probes to ensure data quality during measurements.

Prior to the probe placement, an elastic electroencephalography cap with the traditional “10–20 system” electrode positions marked was placed on the head of each participant to locate the Fp1 and Fp2 areas (Klem et al., 1999). Afterward, the DCS fiber probes with a 2.5 cm source–detector separation were placed on the forehead of the participants over Fp1 and Fp2 positions, thus assessing superficial cerebral cortex areas bilaterally (Figure 3). To ensure probe stability during measurement, an extra fabric layer was applied as tight as possible, considering the participant’s comfort.

Signal quality checks were performed prior, during, and after each session. Initially, to check the device status, the laser power of each probe was measured with a power meter to ensure that laser power complies with the safety standards (<30 mW). After that, an ∼20-min measurement was performed on a stable tissue-mimicking phantom (INO, Biomimic™ Optical Phantoms, Canada) to verify device stability. Once the probes were placed on the participant’s forehead, lasers were switched off and a quick measurement was acquired. The count rate under dark conditions (without laser or external light) should stay below 1 kHz. This step was necessary to ensure probe placement and to scan whether external light was detected. If not, probes were replaced and extra tape and fabric layers were placed on top or made tighter to achieve the desired result.

##### 2.4.2.1 Data pre-processing, motion, and systemic signal regression

In a similar manner to fNIRS, the DCS signal contains physiological and non-physiological noise that could contaminate the data (Tachtsidis and Scholkmann, 2016). Physiological noise includes systemic physiological changes that affect both brain and extra-cerebral tissues, as well as those that do not affect the brain but are included under the probe volume which the DCS cannot separate. These not only include both the potent drivers of CBF, such as the arterial CO_2_ concentration changes, but also “nuisance” parameters such as changes in heart rate, respiratory rate, etc. (Monti, 2011). Non-physiological noise refers mainly to motion artifacts that arise during walking tasks. These need to be regressed out of the signals as is routinely done in fNIRS and fMRI literature. Here, we used a relatively straightforward approach that has been utilized in both fNIRS and fMRI literature (Bowman et al., 2007; Tachtsidis et al., 2010; Gagnon et al., 2011; Kirilina et al., 2012) to de-contaminate the BFI time traces based on secondary measurements using a general linear model (GLM). An abundance of other methods have been proposed to tackle this problem in the fNIRS literature (Patel et al., 2011; Kirlilna et al., 2013; Kiguchi and Funane, 2014; Zhang et al., 2016; von Lühmann et al., 2020), but they have not been yet validated in the context of fDCS and are complex to adapt to our study due to low temporal resolution.

The first pre-processing step was to use the photon count rate to identify periods where the total number of recorded photons would be too low for a reliable analysis. During individual data processing, we reviewed that light intensity was sufficient for good data fitting. The exclusion criteria were: (a) light intensity (I) < 10 kHz; (b) coefficient of variation CV(I) > 5%. The signal was inspected visually for large motion artifacts, e.g., probe misplacement. In that case, data were discarded. The second step used the features of the derived DCS signal to mark periods affected by artifacts such as motion, external light, and simply poor data quality. Then, the standard DCS data analysis methods were used to obtain time traces of BFI (Durduran and Yodh, 2014).

To prepare the data for the GLM model, systemic physiological parameters, i.e., heart rate, respiration rate, end-tidal CO_2_ (EtCO_2_), oxygen saturation (SpO_2_) as well as the accelerometer data were down-sampled through binning to match the timing of the BFI time traces (von Lühmann et al., 2019). All signals were temporally aligned and z-scored. For the z-score normalization, we took into account the mean and standard deviation of each regressor during the whole protocol (Monti, 2011).

For the GLM analysis, the z-scored BFI time trace was used as the dependent variable, while the z-scored systemic and accelerometer data were used as nuisance regressors. The obtained residuals of the single-subject GLM fitting were considered as the regressed BFI vector during the whole protocol period. We assumed that this is a time trace of the cortical CBF that is mainly affected by changes due to the tasks.

To obtain the changes in CBF during each task performed, the regressed BFI vector (*BFI*_*reg*_) was segmented into blocks. Each block included a baseline period (*BFI*_*reg*_(*t*_*baseline*_)^*block*^), 1 min prior to instruction start, the instruction period, data during the task duration, and a recovery period of 1 min after the end of the task. From the whole time trace of each block (*BFI*_*reg*_(*t*)^*block*^) was segmented by the mean value of the data from the baseline period. Finally, the average ΔCBF (Eq. 1) during the whole task period was calculated, where the instruction period was not taken into account.


(1) $$△\,C\,B\,F\,\left(t\right)=B\,F\,I_{r\,e\,g}\,{\left(t\right)}^{b\,l\,o\,c\,k}-B\,F\,I_{r\,e\,g}\,{\left(t_{b\,a\,s\,e\,l\,i\,n\,e}\right)}^{b\,l\,o\,c\,k}$$

This process was repeated individually for each subject. We consider the units of the ΔCBF arbitrary since it refers to the result of the residuals of the GLM model, where all vectors were prior z-scored.

### 2.5 Physical performance assessment

We used the short physical performance battery (SPPB) with balance, gait, and chair stand as sub-items to assess physical function (Guralnik et al., 1994). Gait speed (GS) was calculated from SPPB’s gait item (time required to walk 4 m) as follows: GS = 4/time (m/s). The figure-of-eight (F8T) was used as a more complex gait assessment since it involves a curved pathway as opposed to the straight walkway usually used to calculate gait speed. For the purpose of our study, we only recorded the time and the number of steps needed to walk around the figure-of-eight path around two cones placed 1.5 m apart (Hess et al., 2010).

To further assess the dual-task interference, we performed a 4-m dual-task paradigm separate from the CBF assessments since we anticipated that the long walking distances and instrumentalization during the CBF measures could influence the participants’ usual gait and overestimate the DT impact. To avoid potential learning effects with the counting DT, we chose phonemic verbal fluency (triplets S-A-R and C-P-I) as the cognitive task. The participants were instructed to perform three tasks as follows: (1) verbal fluency single-task, where the participants had to say as many words as possible starting with a predetermined letter for 20 s; (2) walking single-task, where participants had to walk over a 4-m walkway; and (3) dual-task, where participants had to walk 4 m while saying as many words as possible. The order of the tasks and triplets of letters were randomized. Three trials were performed for each task and the mean values of gait speed and the number of words were used for the analysis. For the cognitive component, we calculated word rate (words/s) as follows: number of words/20 s for the single task and number of words/ambulation time for the dual-task. Dual-task costs were calculated as follows: [((dual-task−single task)/single task) × 100].

### 2.6 Cognitive function assessment

We assessed global cognitive function with the MMSE (Folstein, 1975) and frontal function with the symbol digit modalities test (SDMT) (Smith, 1982) and verbal fluency tests (VF) (Patterson, 2011). For the SDMT, which assesses attention, processing speed, scanning, visual speed, and visuomotor coordination (Lezak, 2012), participants wrote down the number corresponding to each symbol according to a displayed key (the score was calculated as the number of correct responses for 90 s). For the Phonemic VF, participants were instructed to say aloud as many words as possible in 1 min starting with a given letter from the triplet P-M-R avoiding proper nouns and words with the same suffix (Lezak, 2012). For the categorical VF, participants had to say as many words as possible belonging to the categories (animals, fruits, …). We provide the adjusted values from the raw scores following regional normative data (Peña-Casanova et al., 2009a,b). We used the Yesavage geriatric depression scale (GDS) as a screening for depressive symptoms (Yesavage et al., 1986).

From the complete neuropsychological evaluation at the outpatient clinic, we collected scores of MMSE, VF, and GDS. Participants in the NC group who did not undergo assessment in the outpatient clinic were assessed by a research assistant (neuropsychologist) under similar conditions. The tests were performed in a quiet room without the fDCS device to provide baseline cognitive function. Only SDMT was performed during CBF measures.

### 2.7 Demographical and clinical characteristics

We collected demographic variables such as age, sex, education, and marital status. As part of a comprehensive clinical evaluation, we recorded the drugs prescribed at the time of enrolment to calculate polypharmacy (5 or more drugs). We collected comorbidities such as hypertension, diabetes, dyslipidemia, arrhythmia, myocardial infarction, heart failure, asthma/chronic obstructive pulmonary disease, epilepsy, stroke, Parkinson’s disease, depression, history of traumatic brain injury, arthrosis, thyroid disease, and sensory impairment. We used the Charlson comorbidity index (Charlson et al., 1994). Data were extracted through an interview with the participant and relatives as well as from medical records.

The ankle-brachial index (ABI) is defined as the ratio of the systolic blood pressure measured at the leg to that measured at the brachial artery (Aboyans et al., 2012). Measurements were performed with the Minidop ES-100VX (Hadeco, Japan) Doppler ultrasound device following previously published recommendations (Aboyans et al., 2012). This measurement was included as a proxy for cardiovascular risk (Criqui et al., 1992).

We assessed functional status with the Barthel index for basic activities of daily living (Mahoney and Barthel, 1965), the Lawton index for instrumental activities of daily living (Lawton and Brody, 1969), and the clinical frailty scale (CFS) (Rockwood, 2005).

### 2.8 Statistical analysis

We performed a descriptive analysis of the global sample. Qualitative variables were described as numbers and percentages. Quantitative variables were described as the median and interquartile range (IQR). Confidence intervals for all analyses were considered at 95%. We performed a bivariate analysis to assess between-group differences relative to cognitive status (NC vs. MCI) with Mann–Whitney *U* test for continuous variables and the chi-square test with Yates’ correction for categorical variables.

The main analysis can be explained in three sections:

1. As the optical measures were repeated within individuals and in the right/left PFC, we performed a linear mixed effects (LME) model to study the changes of CBF across the tests in the dual-task paradigm (NW, FWC, BWC, WWO, and TAP). CBF changes measured from both sides were included in the same LME model to assess the hemodynamic changes in PFC globally and to detect potential modifications in the lateralization of brain activity. In the model, the effects from measures of each test against NW (reference) are presented as fixed effects. The cerebral PFC was measured (left/right) and the participant’s identifiers were treated as random effects. NW was set as the reference, so the estimates from each task were used to assess differences in CBF during FWC, BWC, WWO, and TAP compared to NW within each cognitive status group (objective 1).
2. To assess between-group differences in the CBF pattern (objective 2), an interaction term between task and cognitive status (NC/MCI) was added to the model.
3. Next, we performed separate LME models to assess the effect of several clinical covariates: age, hypertension, diabetes, arthrosis, ABI index, SPPB’s gait speed, and F8T time. These variables were chosen according to clinical relevance or due to differences observed in the bivariate analysis. To avoid the over-adjustment of the model due to the small sample size, we performed a separate model for each variable.

All statistical analyses were performed with the statistical “R” software (*R* version 4.1.3 (2022-03-10), Copyright 2015 The R Foundation for Statistical Computing.

## 3 Results

### 3.1 Sample description

From the initial sample of fifty-four, two subjects were excluded due to low-quality fDCS data, one participant was excluded due to relevant clinical data missing, one participant was excluded due to possible dementia after reviewing clinical records, and one participant could not complete the fDCS evaluation due to technical problems related to the fDCS device. Hence, we included 49 older adults (median age = 78 years, 51% women) who were high functioning in both basic and instrumental activities of daily living, with a low degree of frailty and comorbidity. Gait speed and physical function (SPPB) were slightly above the usual frailty thresholds, with 0.98 m/s and 11 points, respectively. The median MMSE score was 27, with 69.4% (*n* = 34) participants classified as MCI, of whom 38.8% (*n* = 19) had multi-domain and 30.6% (*n* = 15) had single-domain MCI. Table 1 shows the clinical, cognitive, functional, and dual-task performance variables of the sample.

**TABLE 1:** Descriptive of global sample and between-group comparison.

	Study sample *n* = 49	NC *n* = 15	MCI *n* = 34	*P*-value*
**Sociodemographic and clinical variables**				
Age	78 [72, 83]	72 [67.5, 76]	80.5 [73.2, 84]	< 0.001*
Sex (female)	51.02% (25)	46.7% (7)	52.9% (18)	0.92
Marital status (married)	55.12% (27)	80.00% (12)	44.12% (15)	0.04*
Elementary school complete	81.63% (40)	93.33% (14)	76.47% (26)	0.31
CFS score	2 [1.5, 3]	2 [1, 2]	3 [2, 3]	0.002*
Charlson index	0 [0, 1]	0 [0, 0]	1 [0, 1]	0.002*
Hypertension	72.92% (35)	28.57% (4)	91.18% (31)	< 0.001*
Diabetes	20.83% (10)	0.00% (0)	29.41% (10)	0.02*
Dyslipidemia	43.75% (21)	35.71% (5)	47.06% (16)	0.69
Arrhythmia	18.75% (9)	14.29% (2)	20.59% (7)	0.92
Myocardial infarction	12.50% (6)	7.14% (1)	14.71% (5)	0.81
Heart failure	2.13% (1)	0.00% (0)	2.94% (1)	1
Asthma/COPD	12.50% (6)	14.29% (2)	11.76% (4)	1
Thyroid disease	10.42% (5)	14.29% (2)	8.82% (3)	0.96
Traumatic brain injury	14.58% (7)	28.57% (4)	8.82% (3)	0.19
Epilepsy	0% (0)	0% (0)	0% (0)	
Stroke	12.77% (6)	0.00% (0)	18.18% (6)	0.22
Parkinson’s disease	0% (0)	0.00% (0)	0.00% (0)	
Depression	29.17 % (14)	14.29% (2)	35.29% (12)	0.18
Arthrosis	25.94% (18)	33.33% (3)	60.00% (15)	0.32
Number of drugs	4.5 [2.75, 7.0]	2.5 [1.25, 3.75]	6 [4, 7]	< 0.001*
Polypharmacy (5 or more)	50.00% (24)	14.28% (2)	64.71% (22)	0.004*
Ankle-Brachial index	1.16 [1.07, 1.27]	1.18 [1.08, 1.26]	1.14 [1.06, 1.28]	0.6
**Cognitive function**				
MMSE score (0–30)	27 [25, 28]	28 [28, 30]	25 [24.2, 27]	< 0.001*
SDMT PE	10 [5, 11]	11 [11, 13.5]	7 [4, 10]	< 0.001*
Verbal Fluency categorical PE	8 [6, 10]	10 [9, 12]	6 [5, 9]	< 0.001*
Verbal Fluency phonemic PE	9 [7.25, 11.00]	11 [11, 12]	8 [6, 10]	< 0.001*
Yesavage GDS score (0–15)	1 [1, 2]	1 [0, 1.75]	1 [1, 2]	0.09
**Physical performance**				
Barthel index score (0–100)	100 [100, 100]	100 [100, 100]	100 [100, 100]	0.5
Lawton index score (0–8)	8 [7.75, 8]	8 [7.5, 8]	8 [7.25, 8]	0.8
Gait speed (m/s)	0.98 [0.86, 1.09]	1.03 [0.9, 1.17]	0.9 [0.8, 1.1]	0.09
SPPB total score (0–12)	11 [8.25, 12.00]	12 [11, 12]	10 [8, 11]	0.005*
SPPB balance score (0–4)	4 [3, 4]	4 [4, 4]	4 [2.5, 4]	0.038*
SPPB gait score (0–4)	4 [4, 4]	4 [4, 4]	4 [4, 4]	0.4
SPPB chair stand score (0–4)	3 [3, 4]	4 [3, 4]	3 [2.5, 3.5]	0.008*
Figure of eight test time	8.88 [8.03, 11.8]	8.37 [7.60, 8.58]	9.75 [8.12, 12.20]	0.005*
Figure of eight test steps	14.5 [13.0, 17.75]	13 [12, 14]	15 [13, 19]	0.015*
**Dual-task performance**				
Normal walk GS (m/s)	0.97 [0.84, 1.08]	0.99 [0.96, 1.11]	0.91 [0.81, 1.07]	0.1
DT verbal fluency GS (m/s)	0.54 [0.42, 0.62]	0.55 [0.46, 0.64]	0.52 [0.38, 0.59]	0.4
DT cost VF GS (%)	−44 [−50, −35]	−44 [−51, −36]	−44 [−49, −34]	1
ST verbal fluency word rate (word/s)	0.33 [0.27, 0.40]	0.38 [0.34, 0.45]	0.28 [0.25, 0.34]	0.002*
DT verbal fluency word rate (word/s)	0.48 [0.28, 0.64]	0.57 [0.53, 0.69]	0.38 [0.25, 0.56]	0.009*
DT cost word rate (%)	48 [16, 79]	47 [37, 76]	48 [5, 79]	0.7

Values reported are median [Q1, Q3] for quantitative variables and frequencies [% (n)] for categorical variables. *Indicates *p*-value < 0.05 assessed with the Mann–Whitney *U* test and the chi-square test with Yates correction to assess NC vs. MCI between-group differences. NC, participants with normal cognition; MCI, mild cognitive impairment; CFS, clinical frailty scale; COPD, chronic obstructive pulmonary disease; MMSE, mini-mental state exam; SDMT, symbol digit modalities test; BNT, Boston Naming Test; GDS, geriatric depression scale; PE, adjusted scores; SPPB, short physical performance battery; GS, gait speed; DT, dual-task; ST, single-task.

Compared to NC (Table 1), participants with MCI were older, showed higher frailty, polypharmacy and comorbidity levels, and a higher prevalence of hypertension and diabetes. There was no significant difference in other specific comorbidities or the ABI. As expected, participants with MCI showed worse cognitive performance across all the neuropsychological tests, while the Yesavage GDS score was similar between groups. Functional status according to the Barthel and Lawton indices was similar between groups. Participants with MCI had worse physical performance (lower total SPPB score and higher time and number of steps in the F8T), while GS was similar between groups. Regarding the behavioral data during the verbal fluency dual-task paradigm, there was no significant between-group difference in GS during single-task and dual-task walks. Participants with MCI did produce a lower rate of words during single-task and dual-task verbal fluency. The dual-task cost for GS and the number of words were similar across the cognitive status.

### 3.2 Cerebral blood flow changes

#### 3.2.1 Behavioral results during cerebral blood flow monitoring

**FIGURE 4:**
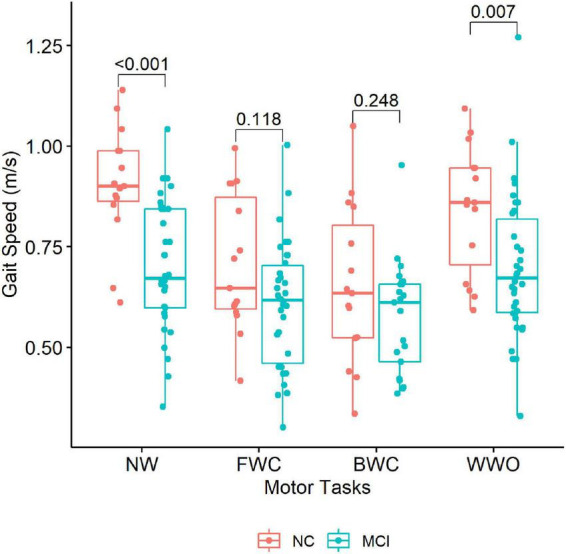
Gait speed across the dual-task paradigm during CBF monitoring. The boxplots depict gait speed during each task stratified by group (NC vs. MCI). *P*-values of between-group differences are indicated with brackets above the boxplots. NW, normal walk; FWC, walk while 2-forward count; BWC, walk while 3-backward count; WWO, walk while negotiating obstacles; NC, participants with normal cognition; MCI, mild cognitive impairment.

Regarding the behavioral data obtained during the CBF measurements, participants with MCI showed lower GS during NW and WWO compared to NC (Table 2 and Figure 4). On the other hand, GS during FWC and BWC was not different between groups. The gait dual-task cost for FWC and BWC was higher among NC, compared to MCI, but showed no differences for WWO.

**TABLE 2:** Behavioral data during CBF monitoring.

	NC *n* = 15	MCI *n* = 34	*P*-value*
NW gait speed (m/s)	0.90 [0.86, 0.99]	0.67 [0.59, 0.84]	< 0.001*
FWC gait speed (m/s)	0.65 [0.59, 0.87]	0.62 [0.46, 0.70]	0.1
BWC gait speed (m/s)**	0.63 [0.52, 0.80]	0.61 [0.46, 0.66]	0.2
WWO gait speed (m/s)	0.86 [0.71, 0.95]	0.67 [0.59, 0.82]	0.007*
DTC FWC (%)	−20 [−3 0, −13]	−10 [−26, −4]	0.03*
DTC BWC (%)	−29 [−34, −18]	−14 [−26, −8]	0.03*
DTC WWO (%)	−4 [−9, −3]	−1 [−6, 2]	0.1
FWC counting errors	0% (0)	20.59% (7)	0.15
BWC counting errors**	26.67% (4/15)	42.86% (9/21)	0.52

Values reported are median [Q1, Q3] for quantitative variables and frequencies [% (n)] for categorical variables. *Indicates *p*-value < 0.05 assessed with the Mann–Whitney *U* test and the chi-square test with Yates correction. **Behavioral data from BWC refers to a sample of 37 (see explanation in Section “3.2.1 Behavioral results during cerebral blood flow monitoring”). NC, participants with normal cognition; MCI, mild cognitive impairment; NW, normal walk; FWC, walk while 2-forward count; BWC, walk while 3-backward count; WWO, walk while negotiating obstacles; DTC, dual-task cost.

Counting errors prevalent during FWC were not statistically different between the two groups.

Twelve participants were not able to perform the BWC task because they could not perform the 3-backward calculation. Those participants did not complete the 5 loops, and hence we did not include BWC-related data in the analysis. Compared to the rest of the sample, participants who were unable to perform BWC all belonged to the MCI group and had higher comorbidity and frailty levels and worse cognitive and physical function (data not shown).

#### 3.2.2 Functional diffuse correlation data

##### 3.2.2.1 Comparison between motor tasks

Among participants with NC, CBF was significantly higher than NW only during BWC (estimate = 0.48, 95% CI [0.21, 0.74], *p* < 0.001) and TAP (estimate = 0.36, 95% CI [0.10, 0.63], *p* = 0.008), but not during FWC and WWO (Table 3 shows LME results).

**TABLE 3:** Linear mixed effects model of CBF changes across the tests in the dual-task paradigm (NW, FWC, BWC, WWO, and TAP).

	Estimate	95% IC	*p*-value
Intercept	–0.08	−0.31, 0.14	0.47
FWC	–0.01	−0.28, 0.25	0.94
BWC	0.48	0.21, 0.74	< 0.001
WWO	0.02	−0.24, 0.29	0.88
TAP	0.36	0.10, 0.63	< 0.01
Cognitive status [MCI] × FWC	0.34	0.02, 0.66	0.03
Cognitive status [MCI] × BWC	–0.04	−0.38, 0.29	0.81
Cognitive status [MCI] × WWO	0.10	−0.22, 0.42	0.54
Cognitive status [MCI] × TAP	0.08	−0.24, 0.40	0.63
Cognitive status [MCI]	–0.02	-0.29, 0.25	0.94
PFC (Right)	0.12	0.02, 0.22	0.02

CBF from both PFCs was included in the model. Normal walk was set as a reference task and an interaction term “cognitive status × task” was added to assess between-group differences. *N* = 49 included in the model. NW, normal walk; FWC, walk while 2-forward count; BWC, walk while 3-backward count; WWO, walk while negotiating obstacles; TAP, heel tapping; MCI, mild cognitive impairment; PFC, prefrontal cortex.

Among participants with MCI, CBF was significantly higher than NW during FWC (estimate = 0.33, 95% CI [0.16, 0.51], *p* < 0.001), BWC (estimate = 0.44, 95% CI [0.23, 0.64], *p* < 0.001), and TAP (estimate = 0.44, 95% CI [0.26, 0.61], *p* < 0.001), but not during WWO.

##### 3.2.2.2 Between-group comparison

###### 3.2.2.2.1 Normal walk to dual-task change

**FIGURE 5:**
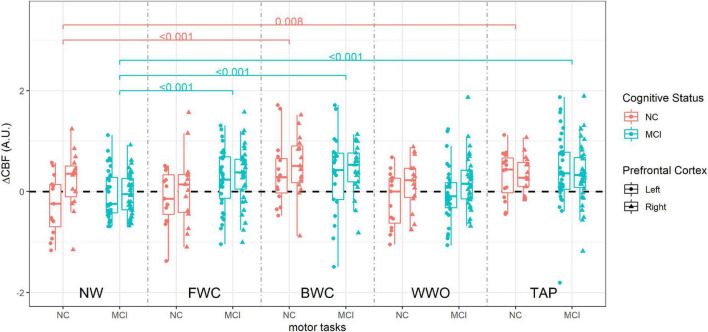
Cerebral blood flow during dual-task paradigm, stratified by group (NC vs. MCI). CBF values from each side are included (right and left prefrontal cortex). *P*-values on brackets indicate between-task CBF comparison within the cognitive status group from the linear mixed effect model. ΔCBF, cerebral blood flow change; A.U., arbitrary units; NW, normal walk; FWC, walk while 2-forward count; BWC, walk while 3-backward count; WWO, walk while negotiating obstacles; TAP, heel tapping; NC, participants with normal cognition; MCI, mild cognitive impairment.

The change in CBF from NW to FWC was significantly higher in MCI compared to NC (estimate = 0.34, 95% CI [0.02, 0.66], *p* = 0.03). CBF change from NW to BWC (estimate = −0.04, 95% CI [−0.38, 0.29], *p* = 0.8) and WWO (estimate = 0.10, 95% CI [−0.22, 0.42], *p* = 0.5) was not significantly different between groups. CBF change from NW to TAP (estimate = 0.08, 95% CI [−0.24, 0.40], *p* = 0.6) was not different either. The PFC measured seems to affect the model with a higher CBF driven by the right PFC (estimate = 0.12, 95% CI [0.02, 0.22], *p* = 0.018). Figure 5 depicts CBF values during each task stratified by cognitive status. See Table 3 for LME results.

###### 3.2.2.2.2 Effect of covariates

The previous model was repeated (one model for each covariate) adjusting for age, hypertension, diabetes, arthrosis, ABI, GS, and F8T time. No significant effect of any of the covariates on CBF was found. See Table 4.

**TABLE 4:** Linear mixed effects models of changes of CBF across the tests in the dual-task paradigm adjusted for clinical covariates.

	Estimate	95% CI	*P*-value model	*P*-value (LRT)*
Age	–0.01	−0.03, 0.007	0.23	0.78
Hypertension	–0.06	−0.39, 0.26	0.69	0.96
Diabetes	0.09	−0.17, 0.35	0.49	0.97
Arthrosis	0.16	−0.056, 0.38	0.14	0.84
ABI	–0.01	−0.34, 0.32	0.95	0.97
GS	–0.23	−0.79, 0.33	0.4	0.8
F8T time	0.04	−0.01, 0.09	0.09	0.9
DTC	0.27	−0.39, 0,9	0.4	0.76

Each row represents one LME model adjusted for each covariate. *P-value (LRT) indicates the comparison of the adjusted model to the original non-adjusted model. ABI, ankle-brachial index; GS, gait speed; F8T, figure of eight; DTC, dual-task cost during verbal fluency.

## 4 Discussion

In our sample of high-functioning older adults, participants with MCI were older and showed higher levels of frailty and comorbidity and worse cognitive and physical performance, while gait performance in a 4-m verbal fluency dual-task paradigm was not different compared to the NC counterparts.

To sum up, both groups had significantly increased CBF during BWC compared to NW, along with a negative impact on gait, while only participants with MCI, the CBF also increased during FWC compared to NW, so the FWC is the dual-task in which we observed a statistically significant difference compared to participants with NC, in particular in the right PFC.

### 4.1 Baseline and clinical characteristics NC vs. MCI

Compared to NC, participants with MCI showed worse physical performance in the total SPPB score and the F8T. We speculate that the curved pathway might be more challenging (Belluscio et al., 2021) for participants with worse motor and cognitive function, as seen in our MCI group. Notably, there was no between-group difference in GS during the single- and dual-task walk. With a quite high GS in both groups during the single task, both groups showed a relevant impact of the phonemic VF dual-task, as shown by a similar GS dual-task cost. Consistent with their worse cognitive function, participants with MCI did produce a lower rate of words during single- and dual-task verbal fluency. Previous studies have reported slower GS during single- and dual-task in MCI compared to controls (Verghese et al., 2008; Montero-Odasso et al., 2012; Pieruccini-Faria et al., 2019); however, dual-task paradigms in the included studies used animal VF and arithmetic tasks (Bahureksa et al., 2017), which limits comparability. The lack of between-group differences in dual-task cost might be due to the similar GS in the single-task and due to the VF dual-task impact seen in the NC group. Our small sample size might have contributed to the findings.

### 4.2 Behavioral data during cerebral blood flow monitoring

Regarding the behavioral data obtained during the CBF measurements, participants with MCI showed lower GS during NW, while GS during FWC and BWC was not different between the groups, so the dual-task cost was higher for NC. In other words, the impact of the arithmetic dual-task seems higher for participants with NC, which could be somewhat surprising. We speculate that participants with NC are better able to perform serial counting, so they allocate more attentional resources prioritizing the cognitive component during the dual-task and thus show a higher impact of the dual-task on gait. Furthermore, a higher GS during NW in participants with NC might give them more room to show a GS decrease. Regarding BWC’s dual-task impact, the twelve participants who were unable to perform the calculation in BWC belonged to the MCI group and showed worse cognitive and physical performance. We cannot rule out that the exclusion of their BWC behavioral results from the analysis might affect the results. These participants might have shown an even slower GS during BWC and could have contributed to a between-group difference in BWC GS. Notably, in this dual-task paradigm, GS in a single task was significantly lower in MCI compared to NC, while we did not find this difference in the verbal fluency dual-task paradigm, as mentioned earlier. This could be due to a fatigue contribution since the measures required longer walking distances or even due to a more challenging walking path since it included turns and instrumentalization due to DCS probes attached to the forehead. Due to the already low GS in NW, a floor effect of GS for the MCI group could contribute to the no difference in GS during the dual-task. Previous studies have reported slower GS in MCI in an arithmetic DT paradigm (Montero-Odasso et al., 2012) and higher dual-task cost in MCI vs. controls (Muir et al., 2012; Pieruccini-Faria et al., 2019). Regarding the cognitive output in the arithmetic dual-task, our study design did not include reporting accuracy rates, so we acknowledge that this limits an appropriate between-group comparison of the counting output during FWC and BWC. The lack of difference in our categorical variable (counting errors) may be also influenced by a low-demanding FWC and the exclusion of twelve participants with MCI who were unable to perform the BWC. Contrary to the findings in the arithmetic dual-task, WWO GS was significantly lower in MCI, and the gait dual-task cost related to WWO was similar between the groups. Our obstacle negotiation protocol might have not been cognitively challenging enough to impact NC’s gait, so the GS during WWO remained slower in MCI compared to NC. Clark et al. (2014) found a slower GS compared to a single-task walk among older adults without cognitive impairment; however, they used 6 obstacles over a 90-m walkway. Coppin et al. (2006) reported slower gait speed in fast-paced obstacle negotiation in participants with poorer executive function.

### 4.3 Functional diffuse correlation data

#### 4.3.1 Summary of comparison of CBF between motor tasks and integration with behavioral results

Cerebral blood flow during BWC and TAP was higher than during NW in both groups, while CBF during FWC was higher than NW only among participants with MCI. We found no differences in WWO-related CBF compared to CBF during NW in both groups.

Among participants with NC, the impact of the arithmetic dual-task on gait seems relevant as seen by a dual-task cost of 20% for FWC and 29% for BWC, but only BWC seems to generate a higher CBF than NW among these participants. On the other hand, among participants with MCI, with a gait dual-task cost of 10% for FWC and 14% for BWC, both FWC and BWC elicited higher CBF than NW. This might be explained by the cognitive component of the arithmetic dual-task. Thus, participants with NC were able to meet the attention load of FWC without increasing the CBF, showing, however, a decrease in GS. On the other hand, participants with MCI required an increased CBF to meet the cognitive load. The fact that CBF during BWC is higher than during NW in both groups goes in line with this explanation. This is further supported by fNIRS studies (Doi et al., 2013; Holtzer et al., 2015), where dual-task broadly show an increase in oxyhemoglobin compared to single tasks, and it is usually interpreted as a response to increasing attention demand.

Our WWO protocol with low small obstacles in fixed positions might have been not challenging enough to elicit an increase in CBF. Evidence from fNIRS studies suggests that a dual-task with obstacle negotiation causes an increase in prefrontal oxygenation compared to a single-task walk in different populations (Clark et al., 2014; Maidan et al., 2016; Chen et al., 2017).

To our knowledge, no previous studies have investigated differences in brain activation between foot tapping vs. overground walking, while foot tapping has been used in fMRI studies (Al-Yahya et al., 2016; Vinehout et al., 2019) or in comparison to motor imaginary (Batula et al., 2017). The study by Al-Yahya et al. did assess fNIRS data during a treadmill walk and fNIRS+fMRI during foot tapping. However, they do not report a comparison of the activation during walk vs. tapping. In our study, TAP caused a higher CBF than NW. This may be due to a higher attention demand of sequential tapping relative to single-task walking or due to a systemic increase in blood flow during TAP (i.e., calves contraction).

#### 4.3.2 Summary CBF change comparison NC vs. MCI and integration with behavioral results

Participants with MCI showed an increased CBF change from NW to FWC relative to NC. We found no between-group differences in CBF change from NW to either BWC, WWO, or TAP. The PFC side had an effect in the model, where differences between MCI and NC in CBF change from NW to FWC were driven by CBF measures from the right PFC.

The CBF change from NW to FWC was significantly higher in MCI compared to participants with NC, even though the FWC’s impact on gait was smaller as seen in the dual-task cost. Hence, the higher CBF change seems due to the cognitive load of FWC in participants with MCI. This is further supported by the fact that a higher CBF was related to the right PFC measures. Functional neuroimaging studies have shown that arithmetic tasks mainly require the activation of frontal and parietal cortical regions, with left-hemisphere lateralization (Burbaud et al., 1995; Kazui et al., 2000; Ueda et al., 2015). Hence, we believe that a higher right activation in MCI compared to healthier counterparts could be explained by the neural compensation theories. According to these theories, older adults with lower brain resources might show an increase in activation as an attempt to maintain performance and even require activation of additional brain regions that can lead to a reduction in hemispheric asymmetry (Reuter-Lorenz and Cappell, 2008; Stern, 2009).

The lack of between-group differences in CBF change from NW to BWC is most possibly influenced by the 12 participants with MCI who were unable to perform the BWC test. These might have shown either higher CBF due to the higher attention load of BWC as a compensation mechanism or lower CBF that would be explained by the capacity limitation theory. Additionally, BWC might be challenging for the NC group too, so the CBF change from NW to BWC required is not different from the MCI group. We believe that WWO and TAP lacked sufficient cognitive load to cause CBF differences between cognitive status groups. Notably, we found no effect of age or any of the clinical covariates on our prefrontal CBF findings. Here, the small sample size may limit the interpretation of the results since it did not allow us to adjust for several covariates and their interactions in the same LME model.

### 4.4 Strengths and limitations

The main strengths of our study consist of a broad measuring protocol with different types of dual-task and a novel approach to study brain activation during motion with fDCS. We provide results from a well-characterized sample, however, of small size, which might have limited our findings. Specifically, the small sample size limited the linear mixed effects model since we were unable to adjust it for several clinical covariates and their interactions to avoid over-adjustment. We acknowledge some methodological issues in our study design: (a) the lack of CBF data during BWC of the participants who were unable to perform the task limits the interpretation of our findings and the comparison of brain activation elicited by the different dual-task; and (b) the lack of an accurate measure of cognitive behavioral results during CBF monitoring, i.e., counting accuracy rate may limit our interpretation of results of the DT interference during the walk while counting paradigm. We provide cognitive output from a separate DT paradigm to assess DT interference in a 4-m usual gait (without the fDCS device), however, with a verbal fluency task to avoid learning effects. The use of a different type of secondary task might limit the extrapolation of results to the counting DT. The low temporal resolution of fDCS is a limitation of our study since not very detailed information can be obtained about the blood flow response during task performance. For the same reason, classic signal regression techniques (e.g., filtering) could not be followed, which led us to use a GLM approach without temporal correlation analysis. We also acknowledge that the eligibility criteria of our study might limit the generalizability of the results.

Overall, to our knowledge, our study is the first to report CBF directly measured during motor tasks with diffuse optics; however, further research is needed to confirm our findings. In the future, fDCS could be combined with fNIRS (Durduran and Yodh, 2014) to elucidate changes in oxygen metabolism.

### 4.5 Conclusion

To sum up, our findings suggest a differential prefrontal hemodynamic pattern in older adults with MCI compared to NC counterparts during our dual-task paradigm. Participants with MCI showed higher CBF related to the dual-task cognitive demand in the easier arithmetic dual-task (2-forward count) compared to older adults with NC. Thus, our results are mainly in line with fNIRS studies, where higher cognitive demands, as in the dual-task, have been related to higher activation (i.e., higher oxyhemoglobin concentrations).

Furthermore, the higher CBF increase from NW to FWC in MCI compared to NC was specifically linked to a right-PFC activation, which we interpret as a compensatory mechanism. Further research is needed to confirm our findings and increase the knowledge about the role of fDCS in neuroimaging to study the efficacy of neural processes in aging.

## Data availability statement

The raw data supporting the conclusions of this article will be made available upon request to the authors, without undue reservation.

## Ethics statement

The studies involving human participants were reviewed and approved by Universitat Autònoma de Barcelona (Barcelona, Spain). The patients/participants provided their written informed consent to participate in this study.

## Author contributions

CU and SA contributed to the conceptualization, data collection, statistical analysis, investigation, methodology, project administration, resources, writing the original draft, and writing, reviewing, and editing. MM-F contributed to the methodology, statistical analysis, writing the original draft, and writing, reviewing, and editing. AR contributed to the writing the original draft and writing, reviewing, and editing. JA and CG-P contributed to writing, reviewing, and editing. LK contributed to the investigation, methodology, and writing, reviewing, and editing. TD and MI the contributed to conceptualization, funding acquisition, methodology, project administration, supervision, writing the original draft, and writing, reviewing, and editing. All authors contributed to the article and approved the submitted version.
